# Comparison of BRICS-TM Countries' Biosimilar Regulatory Frameworks With Australia, Canada and Switzerland: Benchmarking Best Practices

**DOI:** 10.3389/fphar.2021.711361

**Published:** 2021-08-09

**Authors:** Hasumati Rahalkar, Alan Sheppard, Sam Salek

**Affiliations:** ^1^ School of Life and Medical Sciences, University of Hertfordshire, Hatfield, United Kingdom; ^2^ Metina PharmConsulting Pvt. Ltd., Kharghar, Navi Mumbai, India; ^3^ Ascher Resources Ltd., London, United Kingdom; ^4^ Institute of Medicines Development, Cardiff, United Kingdom

**Keywords:** BRICS-TM, ACSS biosimilar, regulatory agency, development, emerging markets

## Abstract

**Objectives:** The aim of this study was to identify, compare and evaluate regulatory requirements for the biosimilar development and review processes in BRICS-TM (Brazil, Russia, India, China, South Africa, Turkey, Mexico) countries with mature regulatory systems of Australia, Canada, Singapore and Switzerland. It is hoped that this benchmark study provides an opportunity for BRICS-TM agencies to identify the key areas for improvement in their regulatory processes.

**Materials and Methods:** A semi-quantitative questionnaire was developed covering the different criteria used in biosimilar development and registration process. Eleven regulatory agencies from BRICS-TM and ACSS (Australia, Canada, Switzerland and Singapore) countries were invited to take part in this study. Data processing and analysis was carried out using descriptive statistics for quantitative data and content analysis to generate themes for qualitative data.

**Results and Discussions:** Nine of the 11 regulatory agencies recruited for the study completed the questionnaire. China and Singapore did not meet the deadline due to lack of resources. The organisational structure of BRICS-TM agencies revealed support from external assessors by most of these agencies in comparison with ACSS agencies. There was absence of reliance approach and participation in harmonisation activities across most BRICS-TM agencies. Despite alignment over biosimilarity, the mandate for *in vivo* non-clinical studies and additional local clinical studies in some of the BRICS-TM countries illustrates a lack of effective implementation of a step-wise approach. Adopting flexible regulatory standards in the sourcing of a RBP (Reference Biologic Product) by BRICS-TM similar to ACSS, will facilitate cost-effective development of biosimilar products.

**Conclusions:** Comparative assessment of the biosimilar regulatory framework of BRICS-TM with ACSS agencies reveals the scope for enhancing efficiency of the regulatory approval process. To achieve this, BRICS-TM agencies should consider relying on reference agencies for alternative review mechanisms such as abridged or verification models, streamlined processes for providing scientific advice to developers and for waiving local clinical studies in-lieu of advanced scientific data.

## Introduction

Developing economies account for one-third of global growth in drug demand, with an overall annual growth rate of 5–8% (IQVIA Institute, 2019). The BRIC (Brazil, Russia, India, China) nations alone account for roughly 30% of GDP globally (Mminele, 2016) along with other emerging markets such as Mexico, Turkey and South Africa (MSCI, 2020).

Opportunities exist for biosimilars in the emerging economies (Boccia et al., 2017), due to low treatment rates with biologics and constraints of affordability. However, a strong and clear regulatory framework is required to unlock the potential of biosimilars in these markets. Despite the BRICS-TM agencies basing their guidelines on a common regulatory framework for Similar Biotherapeutic Products (SBPs) as established by the WHO (WHO, 2013), the biosimilar regulatory requirements, structure and processes are still significantly different. It is therefore challenging to develop biosimilar medicines for simultaneous submission to all the regulatory authorities (Jain et al., 2017). Comparisons of the requirements of regulatory agencies among countries will assist in facilitating improvements in the integration of regulatory processes. Thus, agencies from jurisdictions with emerging pharmaceutical markets might compare themselves with other similarly sized mature regulatory authorities - regulatory authorities associated with an ICH member through a legally-binding, mutual recognition agreement, before October 23, 2015) (WHO, 2021). Also, comparisons between regulatory authorities of similar size, regulatory mandates, structures, resource characteristics and regulatory challenges would be more beneficial than comparisons between authorities with vastly different characteristics and competencies (Mashaki Ceyhan et al., 2018).

Studies have been performed to compare the South African Health Products Regulatory Authority (Keyter et al., 2019), Turkish Medicines and Medical Devices Agency (Mashaki Ceyhan et al., 2018), the Saudi Food and Drug Authority (Hashan et al., 2016) and the Jordan Food and Drug Administration (Haqaish et al., 2017) with the regulatory authorities of Australia, Canada, Singapore and Switzerland, focusing on the area of small molecules.

The aim of this study therefore was to identify, compare and evaluate the biosimilar regulatory strategy of BRICS-TM agencies with that of Australia, Canada, Singapore and Switzerland (ACSS Consortium), in an effort to identify and replicate best practices in biosimilar development and their authorisation processes.

The objectives of this study were to:

• Identify regulatory framework of the ACSS agencies:
- identify resources of the agencies in the biosimilar domain,
- identify biosimilar development criteria i.e., biosimilarity principle, comparative studies including physicochemical characterisation, non-clinical and clinical studies,
- identify the biosimilar marketing authorisation approval pathway specifically for key milestones, validation time, queuing time, backlogs, requirement for sample analysis, conduct of GMP inspections, issuance of Public Assessment Reports (PARs), scientific guidance meetings and clinical trial mandates.
• Identify challenges and areas for improvement.

## Materials and Methods

A semi-quantitative questionnaire was developed for the BRICS-TM agencies based on an already established questionnaire developed by the Centre for Innovation in Regulatory Science (CIRS) (McAuslane et al., 2009) and relevant literature. It was then decided to name this as the: Biosimilar Development, Evaluation and Authorisation (BDEA) questionnaire. For the purpose of this comparison study, it was slightly modified to reflect organisational differences in the regulatory frameworks of Australia, Canada, Singapore and Switzerland regulatory agencies. The rationale for selecting ACSS agencies for the study is because ACSS agencies are like minded agencies and promote work sharing for greater regulatory collaboration and alignment of regulatory requirements. Each of these agencies leverages each country’s strengths, addresses gaps in science, knowledge and expertise and resources to expedite risk assessment, while maintaining or raising quality and safety standards, thereby allowing for rapid assessments to facilitate the market approval of the products (Kühler, 2020). Due to similarity in regulatory systems between these agencies, the ACSS consortium was established in 2007 and now renamed as ACCESS consortium in October 2020 with addition of new agency of MHRA, United Kingdom (ACSS Consortium updated to “Access” which includes United Kingdom as on October 2020) (TGA, 2020). The Consortium builds on international networks, initiatives and mechanisms to advance work- and information-sharing throughout the life cycles of health products (WHO, 2020).

The content validity of the BDEA including its relevance was carried out by a medium sized independent regulatory agency, the Regulatory Authority of Medicines, Equipment and Medical Device (CECMED), Cuba. Post validation, the questionnaire was further concised by removal of duplication of questions and restructured to make a 35-page long questionnaire. The BDEA consists of three parts:

Part I - Organisation of the agency - This part of the BDEA questionnaire consist of current agency structure, resources in the biosimilar domain and types of regulatory review models (Type I- Verification review, Type II- Abridged review and Type III- Full review) employed for scientific assessment, level of data required, and extent of data assessment of the data including reliance on other authorities, if applicable. The rationale for including this section was to assess the capacity, strengths and weaknesses.
Part II – Agency’s guidelines/views on biosimilar development criteria - This part includes questions related to biosimilarity principle, selection of RBP, comprehensive comparability criteria including physico-chemical, non-clinical and clinical studies and “must submit” documents that are required for a biosimilar marketing authorisation application. The rationale for inclusion of this section was to determine the extent of the regulatory requirements for biosimilar development and approval.
Part III – Marketing authorisation approval pathway - This part covers questions with regards to key milestones i.e, the assessment process starting from receipt of the dossier, validation/screening, the number of cycles of scientific assessments including the questions to the sponsor/applicant, expert registration committee meetings to the final decision on approval or rejection of a biosimilar for registration. A standardised process map, developed based on the experience of studying established and emerging regulatory authorities, was embedded in the questionnaire. The rationale for inclusion of this section was to evaluate different stages of the review process and timelines for each milestone.

Eleven regulatory agencies from BRICS-TM and ACSS countries were invited to take part in this comparative study. The study protocol was shared with the 11 agencies together with the electronic self-administered BDEA questionnaire and the supporting instruction for completion. The data collection took place between August to November 2020. The potential study participants were identified via each respective agency’s general email addresses obtained from agency websites, LinkedIn, the research team’s personal contacts, ex-employee and local leading regulatory consultants for each authority. They were selected based on their work experience in the biologic or biosimilar division of the authority, having held a position as a general manager or above (senior management) or a leading regulatory consultant with a close working relationship with the relevant authority in the biosimilar domain. They were sent an electronic mail with brief information about project and the questionnaire, the objective of the study, the number of authorities to be included and requesting their agreement to participate in the study. The questionnaire was completed by a member of the biologic team and approved by the section head. This was followed up by a face-to-face or virtual meetings after receipt of the completed questionnaire with each agency of the BRICS-TM and ACSS countries. Such meetings were arranged to verify the validity of the responses to the questionnaire. Also, copies of the relevant guidelines were requested as part of the questionnaire to verify the responses and to correlate the actual regulatory requirements. In addition, data received from the agency pertaining to number of applications received and reviewed by agencies in reference (ACSS) and test (BRICS-TM) group were assessed.

The therapeutics product branch of HSA (Singapore) was unable to participate due to stretched resources and higher priorities in other areas due to the COVID-19 pandemic. The second-best option of approaching leading regulatory consultant in Singapore was used. However, the participant was unable to provide the necessary information due to lack of time and difficulty in obtaining clarity from HSA on certain areas of the questionnaire.

### Data Processing and Analysis

Data processing and analysis were carried out using Microsoft Excel. The questionnaire (BDEA) is a mixture of qualitative and quantitative questions. Therefore, both quantitative and qualitative analyses were carried out. The descriptive statistics were applied to the quantitative questions of the questionnaire. The analysis for qualitative data was carried out using content analysis and inductive coding in order to generate themes and subthemes (Corbin and Strauss, 1990; Boyatzis, 1998; Thomas, 2006).

### Ethics Issues/Statement

The study has been approved by the Health, Science, Engineering and Technology ECDA, University of Hertfordshire [Reference Protocol number: aLMS/PGR/UH/03332 (1)].

## Results

For the purpose of better clarity, the results will be presented in three parts:

Part I – Organisation of the agency;
Part II – Biosimilar development criteria; and
Part III – Marketing authorisation process.

### Demographic Status of the Study Participants

Out of 11 regulatory agencies (i.e., seven BRICS-TM and four ACSS countries), four BRICS-TM (National Health Surveillance Agency - ANVISA, Brazil; Central Drug Standards Control Organisation - CDSCO, India; South African Health Products Regulatory Agency - SAHPRA; and Turkish Medicines and Medical Device Agency -TITCK) and three ACSS [Therapeutic Goods Administration (TGA) Australia, Biologic and Radiopharmaceutical Drugs Directorate (BRDD) Canada, and Swissmedic, Switzerland] agencies agreed to participate in the study. Since access to two agencies, the Federal Commission for the Protection Against Sanitary Risks (COFEPRIS), Mexico and the Russian Ministry of Health were experiencing resource constraints, a leading regulatory consultant, experienced in working closely with these agencies and having biosimilar expertise was recruited in each country. The respondents from the consulting firms were either the Chief Executive Officer or equivalent. The consultants used for Mexico and Russia were closely associated with the respective agencies regarding registration of biosimilar products, engaged with the agencies for reviewing clinical study protocols and acting as external assessors for the agency relating to biosimilar products. National Medical Product Administration (NMPA), China and Health Science Agency (HSA), Singapore were not able to complete questionnaire on time, due to lack of resources.

### Part I - Organisation of Agency

TGA (Australia) - Though the agency did not have an established dedicated biologic division, the strength of the assigned biologic staff was 3.7% of the total. In addition to qualified internal assessors with B.Sc. to PhD degrees, external evaluators were engaged for the review of clinical data. Of the three review models, “Type II- (Abridged)” and “Type III – (Full review)” of marketing authorisation applications assessments were frequently carried out by the TGA (Table 1).

**TABLE 1:** Comparison of organisational structure and review model.

Criteria	ANVISA	Russia MoH	CDSCO	SAHPRA	TITCK	COFEPRIS	TGA	BRDD	Swissmedic
**Total agency staff**	1600	930	1500	> 200	1172	2000	666	> 10,000	435
**Resource allocation in Biologic/Biosimilar division**									
**Total staff in biologic/ biosimilar division**	24	Not defined	30	10	No information available	20	No biologic division	375	No such division
**Number of biologic/ biosimilar reviewers**	24	Not defined	8	5	No information available	13	25	208	49
**Mean of Applications received (2017–2019)**	10	Not specified	Not specified	Not specified	21	2	6	Not specified	13^a^
**Staff-Workload ratio**	41.7%	NA	NA	NA	Can’t be defined	15.4%	24.0%	Can't be defined	26.5%
**External assessors**									
**Support required**	No	Not specified	Yes	Yes	Yes	Yes	Yes	No	No
**Expertise**	NA	NA	Non-clinical, Clinical	CMC, Non-clinical, Clinical	CMC, Non-clinical, Clinical	CMC, Non-clinical, Clinical	Clinical	NA	NA
**Data Assessment**									
**Review model**	Type III	Type III	Type II, III	Type III	Type III	Type I, III	Type II, III	Type III	Type I, III
**Recognised reference agencies**	Not specified	Not specified	EMA, USFDA, BRDD, MHRA, TGA	Not specified	Not specified	EMA, USFDA, TGA	EMA, USFDA, BRDD, HSA, Swissmedic, MHRA, PMDA	NA	EMA, USFDA

NA: Not Applicable.

a Number of applications received in 2019-data for 2017 and 2018 not specified by the respondent.

BRDD (Canada) - The strength of the biological division within the agency was 2.08% in comparison with the total staff of the agency. The agency restricted use of external assessors and had qualified biological assessors with B.Sc. to PhD degrees. The marketing authorisation applications were reviewed using “Type III - Full review” model with little or no scope for relying on “Type I (Verification)” or “Type II (Abridged review)” models (Table 1). Instead, the agency reviewed applications through the ACSS consortium, based on a work-sharing model.

Swissmedic (Switzerland) - There is no distinct biological division within the agency and hence, with the exception of CMC reviewers, there were common reviewers for reviewing both biologic and non-biologic applications. The minimum qualification of the internal assessors was PhD, PharmD or MD degree. The agency relied on “Type I (Verification)” and “Type III (Full review)” models for biosimilar marketing authorisation applications data assessment.

Comparison of BRICS-TM With ACSS - The strength of biosimilar assessors across BRICS-TM and ACSS was between 1 and 5%, reflecting no large variance in nine agencies resources. Most of the BRICS-TM agencies (except ANVISA and Russia MoH) appointed external assessors to review CMC, non-clinical and clinical data, as compared to ACSS agencies. The ACSS agencies followed the “Type III” model for the majority of the applications and have flexibilities to follow “Type I” (Swissmedic) and “Type II” (TGA) as well. In addition, these four national regulatory agencies have formed the ACSS consortium in 2007 with the objective of enhancing the effectiveness and efficiency of their regulatory systems (TGA, 2020). Some of the BRICS-TM agencies for example India and Mexico partly aligned with the ACSS countries regarding review model, but largely resort to applying Type III (full review) review model. Thus, this indicates that the BRICS-TM countries should not only strive to achieve greater reliance on reviews performed by agencies in their respective region, also to do the same with the established mature agencies.

### Part II - Biosimilar Development Criteria

#### Biosimilarity

The ACSS agencies expected the sponsor to demonstrate biosimilarity of the proposed biosimilar product with its reference product by proving satisfactory physicochemical and biological characterisation with *in vitro* non-clinical PK/PD studies and literature-based clinical performance evaluation, additional *in vivo* safety data plus confirmatory clinical safety and efficacy trials. The TGA, BRDD and Swissmedic accepted clinical data for the Phase III (clinical efficacy) study from reference countries and do not mandate applicant to carry out clinical trials in the local population.

Further, interchangeability is not regulated by law in Switzerland, allowing the prescriber to decide on switching based on patients needs. In the case of TGA, interchangeability policy is under the jurisdiction of the Department of Health and funded through the Pharmaceutical Benefits Scheme. The pharmacists of each province in Canada are charged with the authority to declare two products interchangeable according to its own rules and regulations, without the intervention of the prescriber.

The biosimilarity principles of BRICS-TM agencies are aligned with the expectations of ACSS in terms of different types of studies. The interchangeability decision in BRICS-TM countries lies with the prescriber, except in Russia (where it is regulated by the agency), whereas ACSS follows varied paths. The major challenge with a few of the BRICS-TM agencies is that they require the clinical studies to be conducted locally (Rahalkar et al., 2021), i.e., they do not accept foreign generated clinical data unlike the ACSS agencies (Table 2).

**TABLE 2:** Comparison of biosimilar development criteria of BRICS-TM with ACSS agencies.

Criteria	BRICS-TM agencies	TGA	BRDD	Swissmedic
**Biosimilarity**	✓	✓	✓	✓
Physicochemical and biological characterisation with *in vitro* non-clinical PK/PD studies and literature-based clinical performance evaluation, additional *in vivo* safety data plus confirmatory clinical safety and efficacy trials				
**Interchangeability decision by:**				
Agency	X^a^	X^b^	X	X
Prescriber/physician	✓	X	X	✓
Pharmacist	X	X	✓	X
**Comparative quality characterisation**				
RBP selection				
Must be locally authorised	✓	✓	✓	✓
Acceptance of non-locally authorised markets	EU, US, Canada, Australia, Japan, United Kingdom, Germany^c^ ^,^ ^d^ ^,^ ^e^	EU, US	EU, US, United Kingdom	EU, US
Bridging studies	Not specified	Required	Required	Not required
Analytical specification and method	ICH Q6B (Except Russia MoH, specification same as RBP)	ICH Q6B	ICH Q6B	ICH Q6B
Requirement of comparative stability studies				
Mandatory	✓ (ANVISA, Russia MoH, SAHPRA, COFEPRIS)	X	✓	X
Not mandatory	✓ (CDSCO)	✓	X	X
Supportive	✓ (TITCK)	X	X	✓
**Non-clinical studies**				
*In vitro* studies	✓	✓	✓	✓
*In vivo* studies	✓	X (if addressed *in vitro*)	Case-by-case basis	✓ (as per EMA guideline)
Clinical Studies				
*PK/PD*				
Combined PK/PD study, fingerprinting approach	✓	✓	Not responded	✓
Requirement of immunogenicity studies	✓ (except Russia MoH)	✓	✓	✓
Comparative clinical efficacy studies				
**Clinical study design acceptance**	✓	✓	✓	✓
Equivalence design	✓ (ANVISA, CDSCO, COFEPRIS)	✓	X	X
Non-inferiority design	✓ (Russia MoH, CDSCO, COFEPRIS)	X	X	X
Local clinical studies				

BRICS-TM: ANVISA (Brazil), Russia MoH (Russia), CDSCO (India), NMPA (China), SAHPRA (South Africa), TITCK (Turkey), COFEPRIS (Mexico).

a Regulated by agency in Russia.

b Department of Health.

c TITCK.

d COFEPRIS except United Kingdom, Germany.

e No recognized reference agencies by Brazil, Russia, South Africa.

#### Comparative Quality Characterisation

##### Reference Biologic Product (RBP) Selection

The TGA, BRDD and Swissmedic mandated locally authorised reference products (based on a full dossier including quality, safety and efficacy) that have been marketed for substantial periods of time in their country. They also allowed applicants to use non-locally authorised reference products as part of the development, in the absence of a locally approved reference product. The TGA and Swissmedic accepted use or sourcing of EU or US licensed reference products, whereas BRDD additionally accepted United Kingdom licensed reference products. The evidence of bridging studies to prove sameness of a foreign reference product with the reference product authorised in respective countries was an essential part of the submission for applications to TGA and BRDD. While both agencies required multiple lots of RBP with varied shelf-life for comparability studies, they did not allow change in the reference product during development and comparability studies. The most notable difference was observed with Swissmedic where the requirement for bridging studies had been removed.

While ACSS agencies are flexible for using non-locally authorised reference products, ANVISA and Russia MoH prefer to have locally authorised reference products as part of the development. Although silence on bridging studies (Rahalkar et al., 2018) by each of the BRICS-TM agencies, leads to uncertainty among biosimilar developers, bridging studies could be an unnecessary burden given that if the reference product is the same innovator company, any locally approved product references the same pivotal clinical development data for approval, with changes in manufacturing sites, for example, managed through post-approval changes. It could be deduced that lack of information on bridging studies is in line with good regulatory practices, unless its omission is in contravention of international best practice.

##### Analytical Specification and Method

The TGA, BRDD and Swissmedic followed ICH Q6B (ICH, 1999) for setting the specification considering manufacturer’s experience on SBP and RBP.

The BRICS-TM and ACSS agencies were broadly aligned on this parameter as mentioned in the WHO guidelines (WHO, 2013), except Russia MoH indicating the same specifications for the proposed biosimilar product as those of the RBP by discounting the manufacturer’s experience.

##### Comparative Stability Studies

The TGA recognised the limitations of the biosimilar applicants in matching the age of the proposed biosimilar products with the innovator product and hence did not mandate these studies as part of the application. Swissmedic accepted comparative stability studies as supportive data while BRDD required it as part of the application.

In general, comparability study expectations of BRICS-TM regulatory agencies were similar to those required by the ACSS countries.

#### Non-clinical Studies

The TGA did not require *in vivo* toxicity studies if comparability between the biosimilar and the reference product had been sufficiently addressed by *in vitro* studies based on availability of relevant animal models. Swissmedic followed EMEA/CHMP/BMWP/42832/2005 Rev1 (EMA, 2014) and product-specific guidelines, wherein *in vivo* toxicity studies were required on a case-by-case basis. The requirements for BRDD were unclear.

Unlike TGA and Swissmedic, *in vivo* toxicity study data was essential requirement for the BRICS-TM agencies.

#### Clinical Studies

##### PK/PD

The TGA and Swissmedic both accepted a combined PK/PD study along with a fingerprinting approach. The design, endpoints and fingerprinting approach of BRICS-TM agencies was broadly aligned with ACSS countries.

##### Immunogenicity

All the ACSS agencies indicated the need for comparative immunogenicity as part of the biosimilar application. The data could be obtained from PK/PD studies. The extrapolation of immunogenicity studies to other indications depends on similarity with the RBP or on case-to-case basis. Agencies advised applicants to refer to the EMA immunogenicity guidelines (EMA, 2017) for clarification.

##### Comparative Clinical Efficacy Studies

Clinical study Design - The TGA, Swissmedic and BRDD expected randomised, parallel group, double-blind Phase III clinical trials which are adequately powered using efficacy endpoints unless there are surrogate markers. Equivalence design for the comparative clinical efficacy studies is expected by all the agencies. In addition, TGA also accepted non-inferiority design for the clinical efficacy trials. The clinical study design followed by BRICS-TM agencies is mostly aligned with ACSS countries, with preference over equivalence design. Additionally, ANVISA, CDSCO and COFEPRIS also accepts non-inferiority design for clinical studies.

None of the agencies mandate the clinical studies to be conducted in paediatric or elderly populations for proving comparability of the proposed biosimilar product.

Local Clinical Studies - The ACSS agencies do not mandate clinical trials to be conducted locally in their respective countries.

The PK/PD, immunogenicity and clinical efficacy requirements of BRICS-TM agencies were aligned with ACSS, however the requirement for local clinical studies were unique to Russia MoH, CDSCO and COFEPRIS.

### Part III - Marketing Authorisation Process

#### Scientific Advice

The TGA provides pre-submission advice for the biosimilar application however refrains from providing the same for the development process. Swissmedic and BRDD offers advice during the development of the biosimilar via face-to-face meetings, electronic mail or written responses. The agency advice is not legally binding.

While ANVISA, CDSCO and SAHPRA were aligned with the process of scientific advice, Russia MoH, COFEPRIS and TITCK had yet to develop such communication channels.

#### Clinical Trial Application (CTA) Approval

Swissmedic reviewed the CTA through internal assessors within 30 days and extended it to 60 days if there was a change in the manufacturing process of the biosimilar. Flexibility around the Ethical Committee (EC) letter of submission during evaluation of the protocol existed with Swissmedic. Prior to initiating a clinical trial or implementing an amendment to a clinical trial at a site, BRDD requires the proposed trial protocol and Informed Consent Form (ICF) to be reviewed and approved by a Research Ethics Committee (REC) as defined in the regulations. The TGA does not provide any clarity on the CTA approval process or timelines. Both ACSS and BRICS-TM countries require an Ethical Committee letter submitted during clinical trials.

#### Dossier Review and Approval

The Certificate of Pharmaceutical Product (CPP) in the technical dossier is not required as part of the application by ACSS agencies. The validation of the application and target time to request additional data is in place as one of the milestones. The current queue time with BRDD is 300 calendar days whereas such information was unavailable for the TGA and Swissmedic. External experts for clinical opinion were contractually engaged by the TGA and Swissmedic. The deficiencies in the dossier were presented to the applicant in one single lot from all different sections of the dossier. Sample analysis was performed as part of the market release post approval of the product, in Australia and Switzerland. The GMP inspection is mandated by all the ACSS agencies. For TGA, the GMP inspection could be either on-site or document-based verification and the inspection is conducted concurrently along with the assessment of the product dossier. However, Swissmedic accepted document-based verification for GMP certification of the manufacturing site. The BRDD relies on on-site evaluation for certifying the manufacturer for process and own inspection or pursuant to MOU’s for GMP purposes (Table 3). The on-site evaluation is part of the review process and intends to determine whether a sponsor is “in control” of their manufacturing processes and has suitable QA processes. It is a risk-driven process.

**TABLE 3:** Comparison of dossier review and approval process in BRICS-TM with ACSS agencies.

	BRICS-TM agencies	TGA	BRDD	Swissmedic
CPP requirement	Required	Not required	Not required	Not required
Queue time	28–365 days^a^ ^,^ ^b^	No information^b^	300 days^a^ ^,^ ^c^	No information^a^
Support from external experts	Yes	Yes	No information	Yes
Sharing of queries on dossier to sponsor	As they arise during the assessment^d^ (or)	Collated into a single batch	No information	Collated into a single batch
	Collated into a single batch^e^			
Sample analysis	Before approval	Part of market release	No information	Post approval market surveillance^f^
GMP inspection	On-site (except TITCK)	On-site or document-based verification	On-site evaluation	Document-based verification

BRICS-TM: ANVISA (Brazil), Russia MoH (Russia), CDSCO (India), NMPA (China), SAHPRA (South Africa), TITCK (Turkey), COFEPRIS (Mexico).

a ANVISA, MoH Russia, SAHPRA, TITCK, BRDD, Swissmedic follows calendar days.

b CDSCO, COFEPRIS, TGA follows working days.

c Total review time including submission waiting in queue.

d ANVISA, TITCK.

e Russia MoH, CDSCO, SAHPRA, COFEPRIS.

f On case-by-case basis, possible before approval if any concern regarding quality of the product.

The BRICS-TM agencies (except TITCK) were yet to implement a GMP verification process through off-site review of documents based on a reference agency approval. TITCK issue GMP certificate after paper-based evaluation of the GMP submission.

#### Public Assessment Report (PAR)

Public assessment reports are issued by TGA, Australia (AusPAR) and BRDD, Canada for biosimilar products. Currently, PAR from Swissmedic (SwissPAR) is issued only for new active substances and is available upon request for biosimilars. However, among the BRICS-TM countries, only ANVISA publishes equivalent document on their website. Publication of PARs by ACSS agencies thus ensures transparency, by providing access to information by pharmaceutical industry, other health authorities, healthcare professionals and patients (Papathanasiou et al., 2016), on the approved biosimilar product.

**FIGURE 1:**
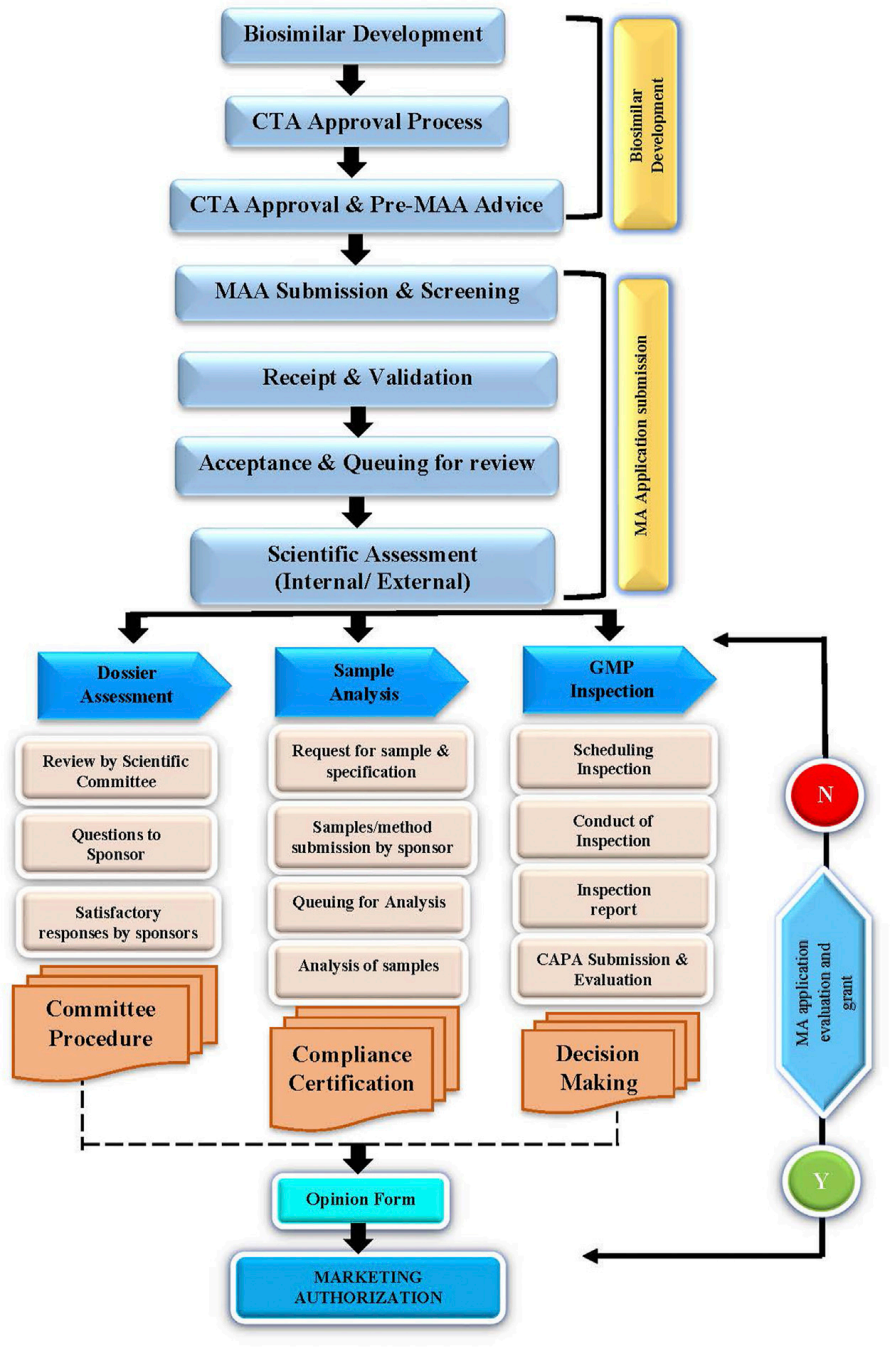
Dossier review and approval pathway.

A flow chart on the dossier review and approval pathway is illustrated in Figure 1.

## Discussion

Biosimilars offer patients in the emerging economies the opportunity to receive key biologic therapies that would otherwise be denied to them due to costs and, therefore, they offer a great growth potential in such economies. However, optimal access to biosimilars depends on collaboration between the relevant stakeholders including policy-makers, regulators, physicians and the industry. In this context, the most important role is played by the regulatory authorities as they provide the regulatory oversight of biosimilars throughout their product life-cycle to ensure only high-quality, safe and effective biosimilars are available in the market (WHO, 2017). However, the regulatory framework for biosimilar development varies in different jurisdictions (Mintz, 2013). In such cases, companies are often required to conduct similar but distinct studies and submit multiple applications for a given product to agencies in different countries (Institute of Medicine, 2013). Duplication of such efforts could have negative impacts on both manufacturers and National Regulatory Authorities (NRAs) (Ball et al., 2016) and this in turn increases the time and cost it takes to bring new drugs to market. Aligning the regulatory strategy across many countries (regulatory harmonisation) could potentially enhance efficiency (WHO, 2017). This will save time and financial resources for drug developers, resulting in earlier access for patients to life saving medicines (Elvidge, 2013).

Comparison of regulatory systems from different countries is one of the methods to gain insights on the limitations of regulatory processes, and thereby to overcome some of these challenges. This study of the guidelines and processes for biosimilar development and authorisation by regulatory agencies in BRICS-TM countries in comparison with the ACSS consortium presents various opportunities to build efficiencies in their respective regulatory frameworks.

Although the organisation is contextual and country-specific often based on country’s legal system, the results for the ACSS countries showed a great similarity (including biosimilarity criteria, RBP selection, setting up specification, non-clinical studies and clinical study requirements). In terms of regulatory requirements being non-contextual, the results confirmed this notion by showing a large degree of similarity. It could be postulated that we could have simply used the acceptable international best practices for purpose of comparison, however, given the dynamic nature of biosimilar development and its expansion, it was envisaged that prospective up-to-date data collection would provide more accurate head-to-head comparison.

Effective implementation of a step-wise approach for demonstration of biosimilarity thereby reducing the need for studies like *in vivo* non-clinical studies and repetition of confirmatory clinical trials in the local population is required (Rahalkar et al., 2021). A policy paper by IGBA (IGBA, 2020) has also emphasized the use of strong analytical science and human pharmacokinetic data for proving quality, safety and efficacy, in-lieu of confirmatory comparative efficacy clinical trials. This science-based evaluation and waiving of comparative efficacy trial has been updated by MHRA, United Kingdom (part of Access consortium) in its updated guidance on the licensing of biosimilar product (MHRA, 2021).

Provision of pre-submission advice and scientific advisory meetings during the biosimilar development and application process to reduce time and costs. Scientific advice (SA) allows early communication between the companies and the regulators. With SA, companies can seek the regulator's opinion on quality, nonclinical, and various clinical aspects (e.g., study design, choice of endpoint, indication) of drug development (Broz et al., 2020; EMA, 2021). Seeking SA on time can support the development of safe and efficacious medicines and ensure that the patients get access to effective treatments in time (EMA, 2021). SA promotes the efficient use of resources as companies receive feedback on viable strategies and methodologies for product development. Companies can plan and design better trials and choose the best endpoints (Dallmann, 2017). By refining the trial design and other aspects as per the SA, companies can save valuable time on prospective queries which may arise during the Clinical Trial Application (CTA) or Marketing Authorisation Application (MAA) (Broz et al., 2020). By fostering scientific collaboration, SA facilitates a working relationship between the company and the regulatory authority. When incorporated into the drug development program, SA can add significant value to the marketing authorisation application. This can significantly enhance the chances of bringing a medicinal product to market (Dokumeds, 2021; Alsager et al., 2015). Allowing applicants to have pre-submission meetings to present the companies product portfolio and discuss overall filing strategies are very much welcomed, especially to discuss products addressing unmet medical need has also been acknowledged by EFPIA (EFPIA, 2017).

The BRICS-TM agencies might have to consider flexibility for using non-authorised reference product from other emerging countries and reference agencies to simplify RBP sourcing. The sourcing of product batches of different ages from different markets for development purposes may present significant difficulties and incur costs (Webster and Woollett, 2017; Rahalkar et al., 2021). In 2009, the World Health Organisation (WHO) Expert Committee on Biological Standardization created a set of recommendations and guidelines to help its member states implement regulation of biologics and biosimilars. However, member states still face regulatory challenges, based on a 2019–2020 WHO survey of participants in 20 countries (Kang et al., 2021) more specifically related to reference biologics, including limited access to information on the reference biologic, financial constraints due to the price of the reference biologic, and difficulty of obtaining reference biologic samples to assess comparability. The authors noted some countries accept reference biologics that are foreign-licensed and -sourced, whereas others require a domestically licensed reference product or bridge studies for a foreign-sourced reference product, which are costly and often result in unnecessary duplication of studies (Rahalkar et al., 2021). Exchanging information with other national regulatory authorities, accepting foreign-sourced reference products, and avoiding unnecessary bridge studies were few of the proposed solutions to address these challenges.

Specifications are critical quality standards that are proposed and justified by the manufacturer and approved by the regulatory authorities as conditions of approval (ICH, 1999). It establishes the set of criteria to which a drug substance, drug product or materials at other stages of its manufacture should conform to be considered acceptable for its intended use. They are linked to the manufacturing process and gives an assurance that the quality is safe and efficacious over its shelf-life. Most of the BRICS-TM agencies are aligned with ICH Q6B, except Russia MoH which expects the specification to of the biosimilar to be same as the reference biologic product. Hence, aligning with the international regulatory standards on setting up specifications based on the manufacturer’s experience with RBP and the proposed biosimilar product becomes an essential aspect to be considered by the agency.

The World Health Organisation (WHO) Certification Scheme was initially implemented to accelerate the availability of new drugs in developing countries by providing evidence of the quality of products through the use of the Certificate of Pharmaceutical Product (CPP) (WHO, 1995; Withing, 2012). However, combined with increased data requirements and review practices of National Regulatory Authority (NRAs), along with the requirement to submit CPP at the time of submission, has delayed the review and approval process and thereby delaying access to patients (Rodier et al., 2020). Also, in a white paper by EFPIA (EFPIA, 2017) on reliance and expedited registration pathways in emerging markets, one of the key points was avoiding non-essential documentation like the request for CPP before approval instead of at time of submission, or to waive the requirement completely. Hence, using alternative data sources such as agency websites for marketing authorisation confirmation instead of requiring CPP as part of the submission needs to be considered by the regulatory agencies of emerging economies like BRICS-TM.

The BRICS-TM agencies should consider acceptance of off-site GMP audit in the GMP accreditation process to reduce delays caused by physical GMP inspections. For instance, the Pharmaceutical Inspection Co-operation Scheme (PIC/S) (PIC/S, 2014) aims at facilitating cooperation and networking between competent authorities, regional and international organisations, thus increasing mutual confidence regarding inspections. Reliance is also an important aspect for conducting desktop assessment of compliance with relevant good practice guidelines and requirements, as described in the respective WHO guidance (WHO, 2018). PIC/S has also issued a guidance on inspection reliance, which outlines a process for the desk-top assessment of GMP compliance (PIC/S, 2018).

Like the ACSS agencies, the emerging economies of BRICS-TM need to move towards reliance and collaboration with other regulatory agencies. A shared or joint review approach for the assessment of dossier in the marketing authorisation application with other comparable agencies and a verification review for products that have been approved by two or more reference agencies and an abridged review for medicines approved by one or more agencies, with a full review only employed for those products that have not been reviewed elsewhere by a reference agency can be considered for reviewing of the product dossier. The World Health Organisation (WHO) supports the implementation of reliance on other regulators work as a general principle in order to make the best use of available resources and expertise. This principle enables leveraging the output of others whenever possible while placing a greater focus at the national level on value added regulatory activities that cannot be undertaken by other authorities, such as, but not limited to, in-country vigilance and market surveillance and control activities and oversight of local manufacturing and distribution. Reliance approaches facilitate timely access to safe, effective and quality-assured medical products and can help in regulatory preparedness and response, particularly during public health emergencies (WHO, 2020).

## Data Availability

The raw data supporting the conclusions of this article will be made available by the authors, without undue reservation.

## Abbreviations

ACCESS, Australia Canada Singapore Switzerland United Kingdom Consortium; ACSS, Australia Canada Singapore Switzerland; ANVISA, Agência Nacional de Vigilância Sanitária; BDEA, Biosimilar Development Evaluation and Authorisation; BRDD, Biologics and Radiopharmaceutical Drugs Directorate; BRICS-TM, Brazil Russia India China South Africa Turkey Mexico; CECMED, Centro para el Control Estatal de Medicamentos Equipos y Dispositivos Médicos; CDSCO, Central Drug Standards Control Organisation; CIRS, Centre for Innovation in Regulatory Science; COFEPRIS, Comisión Federal para la Protección contra Riesgos Sanitarios; CPP, Certificate of Pharmaceutical Product; CTA, Clinical Trial Application; EC, Ethical Committee; GMP, Good Manufacturing Practice; HSA, Health Science Agency; ICF, Informed Consent Form; IGBA, International Generic and Biosimilar Medicines Association; MOU, Memorandum Of Understanding; NMPA, National Medical Product Administration; NRA, National Regulatory Agencies; PAR, Public Assessment Report; PIC/S, Pharmaceutical Inspection Co-operation Scheme; PK/PD, Pharmacokinetics/Pharmacodynamics; QA, Quality Assurance; REC, Research Ethics Committee; RBP, Reference Biologic Product; SA, Scientific advice; SAHPRA, South African Health Products Regulatory Agency; SBP, Similar Biotherapeutic Products; TGA, Therapeutic Goods Administration; TITCK, Türkiye İlaç ve Tıbbi Cihaz Kurumu.
